# Outcomes of primary leadless pacemaker implantation: A systematic review

**DOI:** 10.1111/anec.13084

**Published:** 2023-08-22

**Authors:** Tayyiba Ahmed Noor, Muhammad Omer Rehman Rana, Sapna Kumari, Bakht Umer, Jahanzeb Malik, Amna Ashraf, Maria Faraz, Tabligh Hussain, Muhammad Awais, Amin Mehmoodi, Azmat Hayat

**Affiliations:** ^1^ Department of Medicine Allama Iqbal Medical College Lahore Pakistan; ^2^ Department of Cardiology Chaudhry Pervaiz Ilahi Institute of Cardiology Multan Pakistan; ^3^ Department of Medicine Ross University, School of Medicine Miramar Florida USA; ^4^ Department of Interventional Cardiology Armed Forces Institute of Cardiology Rawalpindi Pakistan; ^5^ Department of Electrophysiology Armed Forces Institute of Cardiology Rawalpindi Pakistan; ^6^ Department of Medicine Military Hospital Rawalpindi Pakistan; ^7^ Department of Business Administration Bahria University Islamabad Pakistan; ^8^ Department of Medicine Ibn e Seena Hospital Kabul Afghanistan

**Keywords:** electrophysiology, implantation, leadless pacemaker, permanent pacemaker, primary pacemaker

## Abstract

**Background:**

During the last decade, leadless pacemakers (LPMs) have turned into a prevalent alternative to traditional transvenous (TV) pacemakers; however, there is no consolidated data on LPM implantation in emergencies.

**Methods:**

Digital databases were searched for this review and four relevant studies, including 1276 patients were included in this review with procedure duration, fluoroscopic time, major complications, and mortality as primary outcomes and pacing threshold, impedance, sensing of LPM, and hospital stay as secondary outcomes.

**Results:**

Gonzales et al. and Marschall et al. showed the duration of the procedure to be 180 ± 45 versus 324.6 ± 92 and 39.9 ± 8.7 versus 54.9 ± 9.8, respectively. Zhang et al. demonstrated the duration of the procedure and fluoroscopy time to be 36 ± 13.4 and 11.1 ± 3.1, respectively. Similarly, Schiavone et al. exhibited intermediate times of implantation at 60 (45–80) versus 50 (40–65) and fluoroscopic times at 6.5 (5–9.7) versus 5.1 (3.1–9). Hospital stay was more with a temp‐perm pacemaker as compared to LPM and pacing parameters were not significantly different in all the studies.

**Conclusion:**

For underlying arrhythmias, whenever appropriate, our review shows that LPMs may be a better option than temporary pacemakers, even as an urgent treatment.

## Graphical abstract

During the last decade, leadless pacemakers (LPMs) have turned into a prevalent alternative to traditional transvenous pacemakers; however, there are no consolidated data on LPM implantation in emergencies. LPMs are a significant contemporary breakthrough in technology in the field of cardiac pacing, and they are feasible in patients requiring emergency implantation of permanent pacing devices. For underlying arrhythmias, whenever appropriate, our review shows that LPMs may be a better option than temporary pacemakers, even as an urgent treatment.

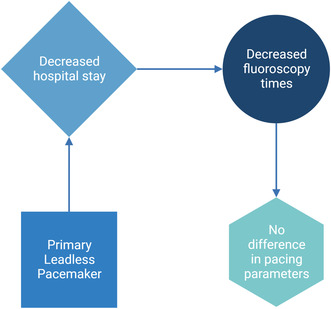

## 1 INTRODUCTION

In the last 10 years, leadless pacemakers (LPMs) have provided an alternative to traditional transvenous (TV) pacemakers (PMs) significantly improving outcomes associated with the need for long‐term pacing (Gonzales et al., 2019; Marschall et al., 2022; Schiavone et al., 2022; Zhang et al., 2021). LPMs have a significantly lower risk of pocket and lead‐related complications frequently seen with TV PMs (Schiavone et al., 2022). LPMs have shown a reduction of 51% of all complications in the first 6 months, with up to 48%–63% at 1 year (Schiavone et al., 2022). The latest evidence exhibited that LPMs have passable parameters during follow‐up, with decreased adverse events (1.77% at 1 year; Ngo et al., 2021).

Adverse events reported with LPMs range between 3.8% and 12.4% and are high in the elderly population (Ngo et al., 2021). First‐generation LPMs only paced the ventricle, limiting their use to atrial fibrillation with block and to patients who contraindication to TV‐PMs (Zhang et al., 2021). The second‐generation LPMs are capable of using VDD pacing, leading to AV synchronous pacing, providing an alternative to patients with atrioventricular (AV) block (Mitacchione et al., 2021). LPMs have bypassed access‐related complications related to TV pacing but do carry their limitations. Although several studies have confirmed these data in an elective setting so far, very little research has been done on the safety and feasibility of LPM implantation in an emergency. Therefore, this review is aimed to investigate the feasibility and outcomes of emergency implantation of LPM in patients referred for urgent PM implantation.

## 2 METHODS

This review was conducted on the Preferred Reporting Items for Systematic Reviews and Meta‐Analyses (PRISMA) statement (Page et al., 2021). Data obtained from the included studies are available in the references section of Table 1.

**TABLE 1:** Study characteristics.

Author (year)	Study design	Males *n* (%)	Sample size (*n*)	Duration of procedure (min)	Fluoroscopy time (min)	Hospital stay (days)	Sensing (mV)	Impedance (Ω)	Threshold (V)	Major compilations (%)	Mortality (%)	Comparator	Predictors for adverse events
Gonzales et al. (2019)	Prospective cohort	8 (78)	LPM = 9 Temp‐perm = 27	180 ± 45 vs. 324.6 ± 92	Not reported	2.3 ± 2.1 vs. 8.5 ± 5.2	Not reported	Not reported	Not reported	11% vs. 15%	11 vs. 11	Temporary‐permanent pacemakers	Not reported
Zhang et al. (2021)	Prospective cohort	3 (37.5)	LPM = 8	36 ± 13.4	11.1 ± 3.1	Not reported	8.3 ± 2.8	788.8 ± 129.2	0.5 ± 0.2	None	None	None	Not reported
Marschall et al. (2022)	Prospective cohort	LPM = 16 (64) Single chamber pacemaker = 28 (52)	LPM = 25 PPM = 53	39.9 ± 8.7 vs. 54.9 ± 9.8	Not reported	Not reported	12.2 ± 10.5 vs. 10.5 ± 8.7	713 ± 191 vs. 669 ± 185	0.4 ± 0.5 vs. 0.6 ± 0.8	Elderly = 0% vs. 5.7% Very elderly = 0% vs. 4.2%	Elderly = 12% vs. 13% Very elderly = 9% vs. 25%	Single chamber pacemaker	Not reported
Schiavone et al. (2022)	Prospective cohort	Emergency = 24 (33.3) Elective = 389 (35.9)	Emergency = 72 Elective = 1082	60 (45–80) vs. 50 (40–65)	6.5 (5–9.7) vs. 5.1 (3.1–9)	7 (3–16) vs. 3 (2–5)	10 (8–12.8)	695 (570–797) vs. 690 (580–810)	0.5 (0.3–0.9) vs. 0.4 (0.2–0.7)	Overall = 6.9% vs. 4.2% Pericardial effusion = 1.4% vs. 0.7% Tamponade = 0% vs. 0.1% Femoral injury = 1.4% vs. 0.6% Device dislodgement = 0% vs. 0.5% Hematoma = 4.2% vs. 2.2%	Not reported	Emergency LPM vs. Elective LPM	Age, BMI, emergency LPM

*Note*: +Where comparators are available, the data are compared with those.

### 2.1 Search strategy and selection criteria

To allocate the studies of interest, we searched databases (EMBASE, PubMed, CINAHL, Web of Science, and Cochrane) with medical subject headings (MeSH) keywords. No language restrictions or time filters were placed; the search strategy and extraction of unidentified articles were performed via backward snowballing (references screening of the relevant articles). The following MeSH terms were used: “Leadless pacemaker” OR “Micra pacemaker” AND “Emergency implantation” OR “Urgent implantation”. The two subsets were combined with Boolean operators. The results from all the combinations were downloaded into the Covidence library for qualitative review.

All titles and abstracts were reviewed independently by two investigators (M.F. and J.M.) who then extracted the articles that reported outcomes of emergency implantation of LPMs, including randomized registries, controlled trials, observational studies, case reports, and research letters. Finally, the analysis did not consider preprints, abstracts, and unpublished data presented at conferences. All data were validated by the senior author (A.H.); in case of missing data, authors of the original article were contacted. The last search ended on January 25, 2023.

### 2.2 Data extraction and quality assessment

The two authors (M.F. and J.M.) extracted the data about LPM implantation in emergencies and its outcomes on patient safety and mortality. Detailed study and patient‐level baseline characteristics, including the type of study, sample size, type of LPM, and patient outcomes were abstracted. Finally, predictors of major adverse events were extracted from each article.

The overall quality was not in the exclusion criteria and the methodological quality of the available studies was performed using the Newcastle‐Ottawa Scale for nonrandomized studies. The quality of the studies is presented in Figure S1.

### 2.3 Statistical analysis

Statistical Package for Social Sciences version 26 (IBM Corp.) was used for statistical analysis. The continuous data were presented as mean and standard deviation while categorical data were expressed with frequency and percentage.

## 3 RESULTS

### 3.1 Search results

**FIGURE 1:**
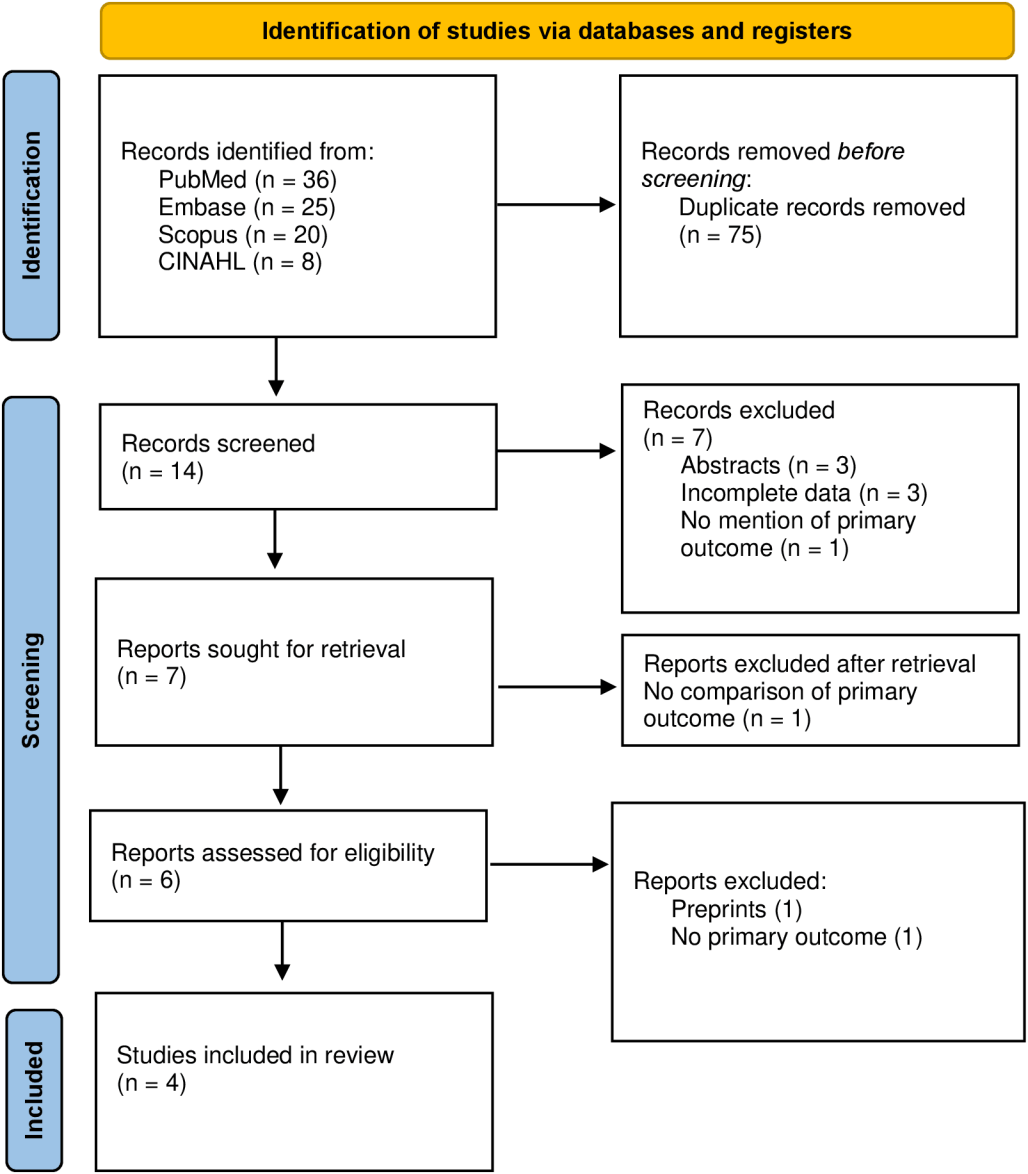
PRISMA flow chart.

The initial search revealed 89 articles. After the removal of duplicates (75) and irrelevant items (7), seven studies were sought for full‐text screens. Of these, three articles were excluded based on different reasons. A total of four studies qualified for qualitative analysis. The PRISMA flow diagram is shown in Figure 1.

### 3.2 Study characteristics and outcomes

In a total of four studies, 1276 patients were included, of which 114 (8.9%) were implanted with LPM and the rest were implanted with either conventional PMs or some other alternatives. In the included studies, 468 (36.6%) patients were males. All four investigations were prospective cohort studies and the included studies were published between 2019 and 2023.

The primary outcomes of our review were procedure duration, fluoroscopic time, major complications, and mortality. The secondary outcomes were pacing threshold, impedance, sensing of LPM, and hospital stay.

### 3.3 Primary outcomes

Of the four studies, one did not compare with any other device, while two studies compared with temporary‐permanent PMs and single chamber PMs. The fourth study compared LPM implantation between emergency and elective cases. Gonzales et al. and Marschall et al. showed the duration of the procedure to be 180 ± 45 versus 324.6 ± 92 and 39.9 ± 8.7 versus 54.9 ± 9.8, respectively, when compared with temporary‐permanent pacemaker implantation. Zhang et al. demonstrated the duration of the procedure and fluoroscopy time to be 36 ± 13.4 and 11.1 ± 3.1, respectively. Similarly, Schiavone et al. exhibited intermediate times of implantation at 60 (45–80) versus 50 (40–65) and fluoroscopic times at 6.5 (5–9.7) versus 5.1 (3.1–9).

Two studies did not report mortality data while Gonzales et al. demonstrated equal mortality among the LPM and temp‐perm group (11% vs. 11%). Marschall et al. showed increased mortality in a very elderly group with implantation of a single chamber pacemaker (Elderly = 12% vs. 13%; Very elderly = 9% vs. 25%).

No complications were reported by Zhang et al. In Gonzales et al., no significant complications were reported in the two groups (11% vs. 15%). In Marschall et al., there were no complications in the LPM group in the elderly and very elderly while the single chamber pacemaker group showed 5.7% in the elderly and 4.2% in the very elderly population. Similarly, in Schiavone et al., the overall complication rate was more in emergency LPM implantation when compared with elective implantation (6.9% vs. 4.2%). Other specific complications are shown in Table 1.

### 3.4 Secondary outcomes

The secondary outcomes for our review, that is, hospital stay were reported in two studies, and pacing parameters were shown in three studies. Hospital stay was more with a temp‐perm pacemaker than LPM (8.5 ± 5.2 vs. 2.3 ± 2.1) in Gonzales et al. In Schiavone et al., the emergency LPM implantation had higher days of hospital stay when compared with elective procedures (7 (3–16) vs. 3 (2–5)). Pacing parameters were not significantly different in all the studies (Table 1).

## 4 DISCUSSION

This study represents the first focused analysis of LPM implantation given as therapy for emergency use of pacemaker indications. In this systematic review of four observational studies, we explored the feasibility and complication rates of urgent LPM implantation, as compared with other devices or elective LPM implantation. The main findings of our study can be summarized as follows: (i) LPM implantation is a feasible option for urgent implantation for the treatment of severe bradyarrhythmias as an urgent treatment strategy, (ii) LPM implantation was correlated with decreased mortality and a decreased rate of major complications, (iii) LPM implantation demonstrated low procedural times, hospital stay, and fluoroscopy time but one study demonstrated more procedure time in an urgent setting, and (iv) pacing parameters were comparable in both comparison with other cardiac implantable electronic devices and elective LPM implantation.

TV‐PMs are an established treatment option for the treatment of bradycardia, even in emergency implantations. In previous years, LPMs have evolved into the contemporary form of permanent pacing therapy, particularly in patients requiring single‐chamber PMs. Observation of device‐related complications, including lead fractures, pocket and lead infections, and venous complications has led to the advent of LPMs and defibrillator systems. The LPM has numerous advantages over TV‐PMs, especially in patients undergoing lead extractions. It confers a lower risk of device infection, it can avoid the pocket formation and venous access. In selected patients, an LPM can be considered an alternative to a TV‐PM when continuous pacing support is necessary for the patient.

Studies included in this review suggest that LPMs are tolerated well in all age groups, including the elderly and the very elderly. Previous studies have shown concerns about lead perforation in elderly patients with LPM implantation. Therefore, LPMs have been generally restricted to a highly selected group of patients, even in elective settings. However, given the cumulative results of this review, we believe that all patients with urgent indications for pacemaker implantation might benefit from LPMs rather than other devices or temporary PMs. The overall complication rate is less when compared with other devices, despite the effect of the learning curve of the operators involved.

At present, no randomized trials have directly evaluated the performance and safety of LPM with conventional devices and all data were derived from observational studies using historical cohorts of conventional devices. In a recent study, the results of the longitudinal coverage with evidence development study on the micra leadless pacemaker (Micra CED) demonstrated the safety profile of LPM, compared with traditional devices (El‐Chami et al., 2022). No difference in the 30‐day complication rate was observed between the two groups. Analysis of 6‐months complication rates, LPM fared better when compared with TV‐PM.

Historically, the pacing mode available in PLM was VVI‐R, thus indication for the use of LPM was limited to patients with atrial fibrillation with concomitant atrioventricular block, patients with sinus node dysfunction, and geriatric age‐group patients with a low level of mobility. However, after the advent of contemporary LPMs, implantation of these devices may be considered in patients with AV block. AV synchrony can be achieved by the accelerometer used for rate adaptiveness which can also track atrial mechanical activity. The micra accelerometer sensor sub‐study (MASS and MASS2) showed the feasibility of atrial mechanical activity tracking and confirmed the efficacy and safety of this algorithm (Chinitz et al., 2018).

The potential limitations to this review were as follows: (i) there was a paucity of literature on our review question and the only available studies were observational cohort studies, limiting the cause‐and‐effect relationship of LPM and complications, (ii) the experience of different operators in all included investigations cannot be assessed and this may have introduced a selection bias in patient groups, and (iii) the sample size was too small for a quantitative review and larger observational studies and randomized controlled trials are needed to evaluate safety and efficacy of LPMs in emergency settings.

## 5 CONCLUSION

LPMs are a significant contemporary breakthrough in technology in the field of cardiac pacing, and they are feasible in patients requiring emergency implantation of permanent pacing devices. For underlying arrhythmias, whenever appropriate, our review shows that LPMs may be a better option than temporary PMs, even as an urgent treatment.

## AUTHOR CONTRIBUTION

Concept: MF, JM, AM. Methodology: MORR, TAN, SK, BU. Lit search: AA, MF, TH, MA. Writing: JM, MF, MA, AH, AM, BU. Supervision: AM, AZ.

## CONFLICT OF INTEREST STATEMENT

The authors have no competing interests to declare.

## ETHICS STATEMENT

No ethical review was necessary for this review as no human participants were analysed.

## Supporting information


Figure S1

## Data Availability

Data sharing not applicable to this article as no datasets were generated or analysed during the current study.
