# Supplementary figures and images for: Outcomes of primary leadless pacemaker implantation: A systematic review
Source: Ann Noninvasive Electrocardiol. 2023 Aug 22;28(6):e13084. doi: 10.1111/anec.13084 (PMC10646376; doi:10.1111/anec.13084)

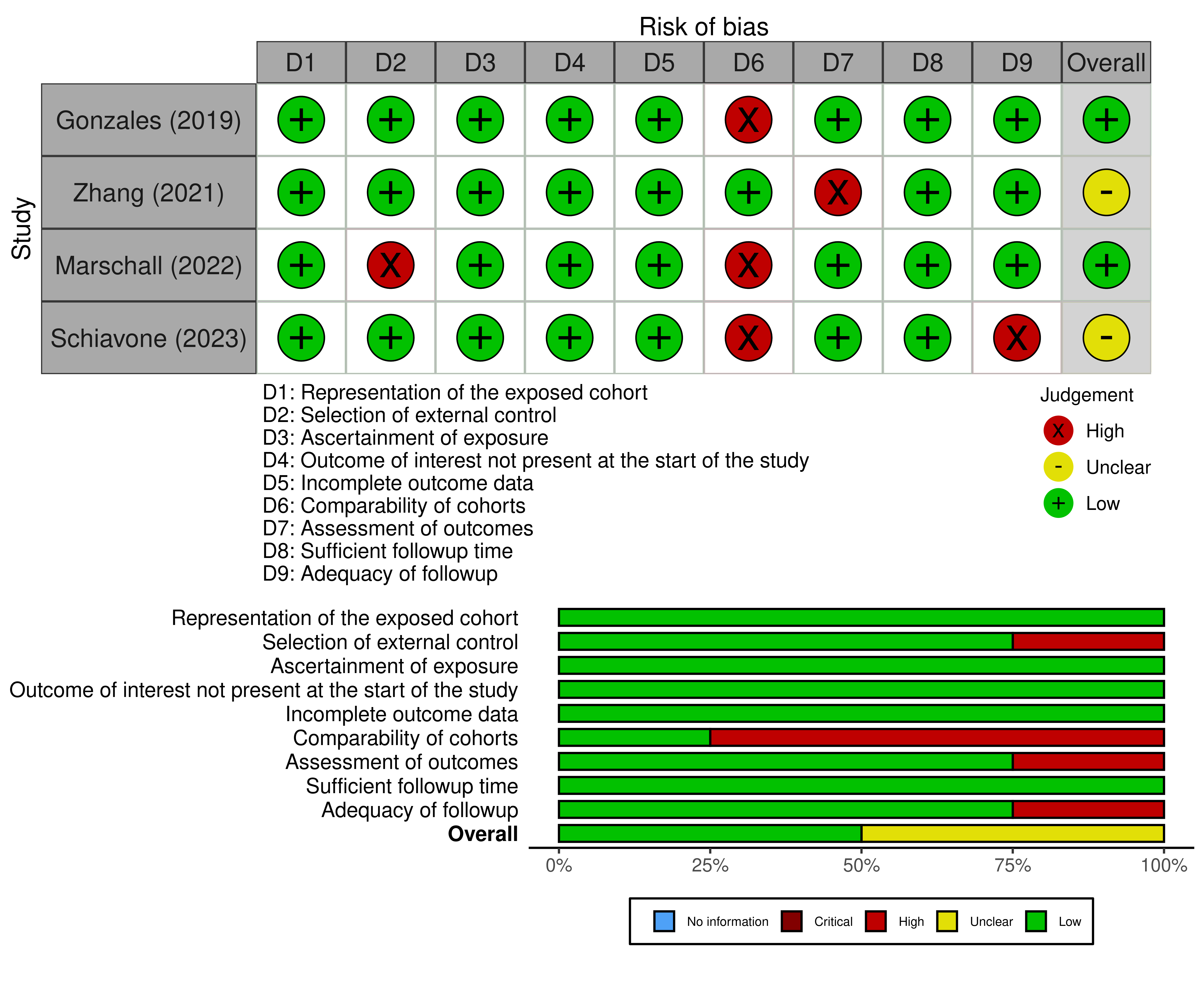

Supplement: Supplementary file 1 — Figure S1 [file ANEC-28-e13084-s001.png]
